# Abnormal Typhoid

**Published:** 1898-08-06

**Authors:** 


### ABNORMAL TYPHOID.

A very interesting case is reported by Dr. Sidney Herbert Snell[1] in which a prolonged pyrexia, lasting between four and five weeks, ended in perforation of the intestine and death. The patient was a man aged 45 years, who had always been of quiet, temperate habits, living a plain, semi-country life. After being out of sorts for a little more than a week, even prior to which he had not felt quite well for some weeks, he was one day driving for some time in heavy rain although he did not seem to have been really wetted. That night he had a sharp attack of diarrhœa. Three days after that he was complaining of general malaise with slight headache, he looked "livery," but there was no actual jaundice and no fever. The bowels were acting once a day, somewhat light in colour, and with a little slime. Two days later there was slight pyrexia. The next day, *i.e.*, a week after the drive, the pyrexia had increased, and it continued to increase for several days, after which it fell again, and continued for the rest of his illness between 99 deg. and 101 deg. Debility, quick pulse, and this almost constant pyrexia were practically the whole of the symptoms for nearly a month, when the temperature fell to normal, but this was quickly followed by extreme weakness, a rise of temperature, very rapid pulse of a quickly collapsing character, wandering, and an anxious and pinched appearance, and four days after this exacerbation of the symptoms death occurred. From the first the possibility of the case being one of typhoid fever was kept in mind, and for a time that seems to have been the diagnosis which was favoured. Sir William Broadbent, however, who adds a note on this case, says, "The state of the tongue was the reverse of characteristic; the abdomen was normal in appearance, and everywhere soft and supple; there was no enlargement of the spleen, but the liver could just be felt." The motions also were not characteristic. "Although, therefore, the investigation was begun under the idea that the continued fever of three weeks' duration was probably enteric, I came to the conclusion that this was not the case here. It seemed to me also that tubercle could be excluded, and the only diagnosis at which I could arrive was that there was internal sepsis of some kind, not specifically enteric or typhoid." On a post-mortem examination being made by Dr. Hugh R. Smith, purulent peritonitis was discovered, which had evidently been caused by three perforations in the small intestine between twelve inches and sixteen inches above the cæcum. These, however, were not of the usual character or in the usual situation, nor were Peyer's patches infiltrated. On a bacteriological examination of the spleen being made by Dr. E. D. W. Greig the bacillus coli communis was found to be present in a pure state and in a highly virulent condition. "It is fortunate," says Sir William Broadbent, "that bacteriological examination could come to the aid of clinical observation and morbid anatomy. There would otherwise have been no escape from the conclusion that typhoid fever could assume a type so aberrant as to be unrecognisable clinically, and could give rise to lesions quite unlike those usually met with in this disease. It would be premature to assume that the bacterium coli commune rendered specially virulent by unknown conditions was responsible for the train of symptoms and for the lesions met with in this patient, but it seems highly probable that such was the case." The fact is, that we know far too little of the vagaries of the bacterium coli, a micro-organism which is almost ubiquitous in the busy haunts of man, and, in general, seems practically harmless, but yet is endowed with the property of sometimes invading the tissues and sometimes becoming highly virulent.

The important point in this case, however, is the fact that what might have been taken as a merely abnormal case of typhoid, and might then have been used to show how far typhoid fever might depart from what is typical, both in regard to symptomology and morbid anatomy, has been shown not to have been a case of typhoid at all. By many practitioners typhoid fever is used as an all-embracing term, a large umbrella under which all sorts of lingering pyrexias which recover may find shelter. Perhaps they may be right; nevertheless, it must be insisted on that the symptomology of the disease must be worked out, not from recoveries, but from the histories of fatal cases proved post-mortem. On the other hand every well-observed case, such as the one we have referred to, in which an apparently "causeless" pyrexia can be traced to some other source than a hypothetical atypical typhoid, widens the field in which we may search for the origin of such conditions and lessens our excuse for believing, as some people are ready to say, that typhoid will account for anything. The more we can explain away cases of so-called "abnormal typhoid" by proving that they arise from other causes, the more definite becomes the meaning of the term typhoid fever.

[1] *Lancet*, July 30.

